# Nursing Resources Linked to Postsurgical Outcomes for Patients With Opioid Use Disorder: An Observational Study

**DOI:** 10.1097/AS9.0000000000000185

**Published:** 2022-07-22

**Authors:** Rachel French, Matthew D. McHugh, Linda H. Aiken, Peggy Compton, Salimah H. Meghani, J. Margo Brooks Carthon

**Affiliations:** From the * Center for Health Outcomes and Policy Research, Center for Mental Health, and National Clinician Scholars Program, University of Pennsylvania, School of Nursing & School of Medicine, Philadelphia, PA; † Center for Health Outcomes and Policy Research, University of Pennsylvania, School of Nursing, Philadelphia, PA; ‡ University of Pennsylvania, School of Nursing, Philadelphia, PA.

**Keywords:** nurse education, nurse staffing, nursing resources, opioid use disorder, postsurgical outcomes

## Abstract

To determine whether better nursing resources (ie, nurse education, staffing, work environment) are each associated with improved postsurgical outcomes for patients with opioid use disorder (OUD).

**Background:**

Hospitalized patients with OUD are at increased risk of adverse outcomes. Evidence suggests that adverse postsurgical outcomes may be mitigated in hospitals with better nursing resources, but this has not been evaluated among surgical patients with OUD.

**Methods:**

Cross-sectional (2015–2016) data were utilized from the RN4CAST-US survey of hospital nurses, the American Hospital Association Annual Survey of hospitals, and state patient hospital discharge summaries. Multivariate logistic and zero-truncated negative binomial regression models were employed to examine the association between nursing resources and 30-day readmission, 30-day in-hospital mortality, and length of stay for surgical patients with OUD.

**Results:**

Of 919,601 surgical patients in 448 hospitals, 11,610 had identifiable OUD. Patients with compared to without OUD were younger and more often insured by Medicaid. Better nurse education, staffing, and work environment were each associated with better outcomes for all surgical patients. For patients with OUD, each 10% increase in the proportion of nurses with a bachelor’s degree in nursing was associated with even lower odds of 30-day readmission (odds ratio [OR] = 0.88; *P* = 0.001), and each additional patient-per-nurse was associated with even lower odds of 30-day readmission (OR = 1.09; *P* = 0.024).

**Conclusions:**

All surgical patients fare better when cared for in hospitals with better nursing resources. The benefits of having more nurses with a bachelor’s degree and fewer patients-per-nurse in hospitals appear greater for surgical patients with OUD.

## INTRODUCTION

Over 10 million people in the U.S. report misusing opioids, with 2 million living with opioid use disorder (OUD).^1^ These people will likely need hospital care including elective and emergent surgery, and when they do, will need tailored care to meet their needs.^2^ An estimated 4%–11% of the 36 million patients hospitalized annually in the United States have OUD,^3^ and these patients are at increased risk of morbidity, mortality, and high healthcare-related costs.^4,5^ Surgical patients with OUD, on average, are in the hospital for 2 more days (6 vs 4 days)^5^ than those without OUD, and evidence among orthopedic surgical patients suggests they have nearly 4 times higher odds of dying during their hospitalization.^6^ Despite being younger and often having fewer comorbidities,^4^ surgical patients with OUD also experience a 46% higher risk of 30-day and 15% higher risk of 90-day readmission compared with surgical patients without OUD.^7,8^

Differences in outcomes for patients with OUD may be related to a number of factors, including distinct postoperative care challenges, such as achieving adequate pain and withdrawal management that, left untreated, can prompt a patient-directed discharge (discharge against medical advice).^2,9–11^ Nurses are key to facilitating adequate pain management, care-team collaboration, patient assessment, and patient teaching postoperatively, all of which are particularly salient for patients with OUD.^2^ An inadequate supply of nursing resources impacts clinical care delivery, and these deficits may differentially impact vulnerable subpopulations of surgical patients, including those with OUD.

Nursing resources, such as nurse education (proportion of nurses with at least a Bachelor of Science in Nursing [BSN] degree), nurse staffing (the ratio of patients-to-nurses), and the nurse work environment (organizational context that facilitates high-quality care delivery), ensure that nurses can address the specific needs of patients with OUD and work with other clinicians to ensure that patients with OUD receive the services they need during hospitalization. In addition to a large body of evidence substantiates that a wide range of surgical patient populations have better outcomes when cared for in hospitals with better nursing resources (eg, higher proportions of nurses with BSN degrees, fewer patients-per-nurse),^12–15^ research also suggests that at-risk populations undergoing surgery, such as those with serious mental illness^16^ and those with Alzheimer’s disease,^17^ particularly benefit from receiving care in hospitals with better nursing resources. Evidence also suggests some minoritized populations (eg, older Black adults) experience greater benefit when cared for in hospitals with greater nursing resources.^18^ Building on the robust evidence base linking better nursing resources to improved postsurgical patient outcomes,^12–14,16–22^ we sought to determine whether nurse education, staffing, and work environment were associated with key postsurgical outcomes (ie, 30-day readmission, 30-day in-hospital mortality, and length of stay) for patients with OUD.

## METHODS

### Study Design

This cross-sectional study leveraged data from 3 linked datasets from 2015 to 2016 in 4 states (California, Florida, New Jersey, and Pennsylvania). Data were linked by a common hospital identifier from (1) the RN4CAST-US survey of hospital nurses, (2) the American Hospital Association Annual Survey of hospitals, and (3) state patient hospital discharge summaries. The relationships among nursing resources and patient outcomes were examined for patients with OUD, controlling for patient- and hospital-level covariates. This study was approved by the University of Pennsylvania’s Institutional Review Board.

### Data and Sample

#### Hospitals

The 2016 American Hospital Association Annual survey provided hospital characteristics including bed size, teaching status, technology status, urban status, and state. Data on hospital nursing resources were derived from the RN4CAST-US survey. This survey was conducted using a modified Dillman^23^ approach and was sent via postal or electronic mail (addresses obtained by state boards of nursing) to a 30% random sample of licensed registered nurses in California, Florida, New Jersey, and Pennsylvania between 2015 and 2016. The response rate of the nurse RN4CAST-US survey was 26%^24^ and respondents represented more than 95% of hospitals in the 4 states.^22^ To address potential concerns about nonresponse bias at the nurse level, a second random sample of 1400 nonrespondents was conducted. Intensive recruiting methods were employed to incentivize participation in the survey resulting in a response rate of 87%. Representativeness was compared to the study sample and no response bias was found.^24^ Respondents to the RN4CAST-US survey were asked to list the name and address of the hospital in which they worked. These responses were aggregated to the hospital level to derive hospital-level measures of nurse education, staffing, and work environment. Additional information on this survey is available.^22,24^

**FIGURE 1.**
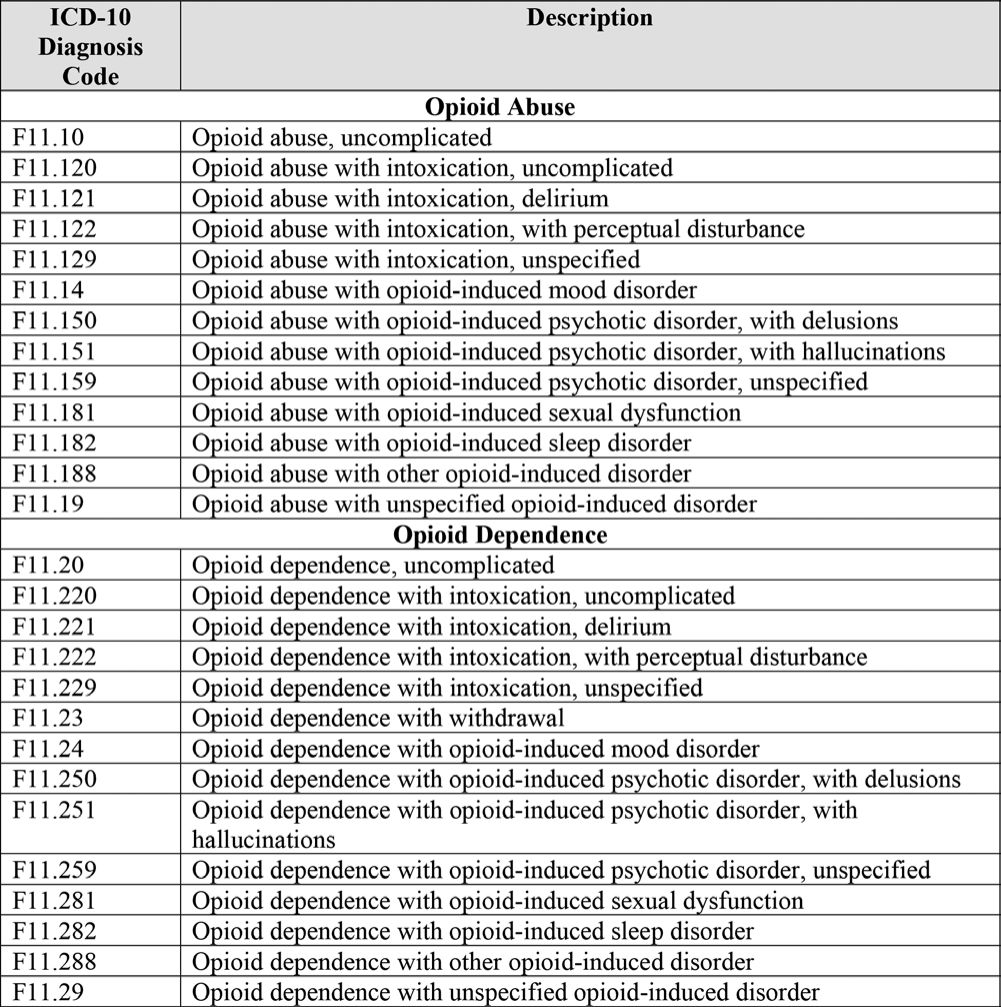
ICD-10 diagnosis codes for OUD. This figure shows the ICD-10 codes used to classify OUD for this study.

We limited analysis to hospitals that had at least 10 nurse survey respondents.^14^ On average, hospitals had 61 nurse survey respondents (median = 48), ranging from 10 to 204 nurses. Hospitals were included if they had at least 150 surgical patient discharges to ensure adequate patient volume^25^ and 5 patients with OUD to ensure that hospitals had experience treating patients with OUD.^26^ Only patients with opioid abuse or dependence not in remission based on International Classification of Diseases, Tenth Revision (ICD-10) codes were identified as having OUD (Fig. 1). The average number of surgical patients was 2053 (median = 1759; range = 193–10,909) and of surgical patients with OUD was 26 (median = 17; range = 5–546). The final sample included 488 nonfederal, acute care hospitals in California, Florida, New Jersey, and Pennsylvania.

#### Patients

De-identified patient data from 2015 and 2016 were derived from annual inpatient discharge summaries from the California Office of Statewide Health Planning and Development, the Florida Agency for Health Care Administration, the New Jersey Department of Health and Senior Services, and the Pennsylvania Health Care Cost Containment Council. Patient diagnostic codes for identifying OUD, surgical procedure, and comorbidities, as well as demographic characteristics, including sex, race, ethnicity, and insurance status, were obtained from these data sources. These data sources were used to generate the outcomes of interest. Patients were included in the study sample if they were admitted to a study hospital in the fourth quarter of 2015 or any quarter of 2016 because that time period overlapped with ICD-10 coding, which may capture OUD stays that were missed by the former International Classification of Diseases, Ninth Revision codes.^27^ Patients were 18 to 99 years old and underwent general, orthopedic, or vascular surgery. These procedures were selected because they allow for well-validated risk adjustment and almost all acute care hospitals perform them.^18,21^ Only the index admission was evaluated for each patient.

### Study Variables

#### Patient Outcomes

Outcomes included 30-day readmission, 30-day in-hospital mortality, and length of stay. Readmission was defined as rehospitalization within 30 days of discharge from the index hospitalization. Only patients who were discharged alive were included in readmission analyses.^14^ Mortality was identified if a patient died in the hospital within 30 days of admission. Length of stay was calculated based on the number of days a patient spent in the hospital during the index hospitalization.

#### Hospital Nursing Resource Variables

The main predictor variables were proportion of nurses with a BSN, patient-to-nurse staffing ratios, and the nurse work environment. They were measured at the hospital-level. A measure of nurse education was generated such that each unit increase equated to a 10% increase in the hospital-proportion of nurses educated at the BSN level or higher. Consistent with existing literature,^22,28^ we constructed a hospital-level measure of nurse staffing by dividing the number of patients by the number of registered nurses on each unit during the last shift and aggregating nurse responses to the hospital level. The nurse work environment measure was derived from the Practice Environment Scale of the Nursing Work Index-Revised (PES-NWI), a validated, 31-item measure.^20,29^ The PES-NWI has 5 subscales (Nurse Participation in Hospital Affairs; Nursing Foundations for Quality of Care; Nurse Manager Ability, Leadership and Support of Nurses; Staffing and Resource Adequacy; and Collegial Nurse-Physician Relations)^29^ that were each aggregated to the hospital-level. Hospitals were classified as having poor, mixed, or good work environments corresponding to the bottom 25%, middle 50%, and top 25%, respectively, of the composite PES-NWI score distribution. These approaches for measuring nursing resources have been well-established and validated in the literature.^15,21,30^

#### Covariates

##### Patient Characteristics

Patient covariates included age, sex, each Elixhauser comorbidity^31^ (except drug abuse^5^), and each Medicare Severity Diagnosis-Related Groups. Our interest in covariate selection was in controlling for confounders to best isolate the relationship between variables of interest and outcomes. Accordingly, race, ethnicity, insurance status, and discharge disposition were included as covariates. These factors are distributed differently across surgical patients with OUD compared with those without.^4,5^ They are also correlated with the outcomes of interest in this study.^32^ Specifically, race and ethnicity are associated with differences in postsurgical outcomes,^18,33^ with most evidence suggesting that Black patients are at higher odds of readmission compared with White patients.^34–36^

##### Hospital Characteristics

Hospital covariates included bed size, teaching status, technology status, and urban status. Hospitals were classified as small (100 beds or fewer), medium (101–250 beds), or large (more than 250 beds). Teaching status was categorized as nonteaching (no medical residents), minor teaching (1:4 or smaller trainee-to-bed ratio), and major teaching (higher than 1:4 trainee-to-bed ratio). Hospitals were classified as high technology if they had facilities for open-heart surgery, major organ transplants, or both. Urban status was based on Core-Based Statistical Area^37^ codes.

### Statistical Analysis

We described patient characteristics and outcomes among surgical patients, and differences between patients with and without OUD, in the sample using ANOVAs, χ^2^ tests, and *t* tests. We also described hospital characteristics, as well as hospital-level measures of nursing resources and number of patients with OUD. A series of logistic (for readmission and mortality) and zero truncated negative binomial regression (for length of stay because a zero value cannot occur^38^) models were used to estimate the relationships between each nursing resource and postsurgical outcomes.

The first model tested the unadjusted effect of having OUD and each nursing resource (separately) on outcomes. The final models addressed our primary objective: to determine whether better nursing resources (ie, higher proportions of nurses with BSN degrees, fewer patients-per-nurse, higher ratings of the nurse work environment based on the PES-NWI) were associated with postsurgical outcomes for surgical patients with OUD while controlling for patient and hospital characteristics. In each of these models, the results represent the change in outcomes (odds ratios [ORs] for readmission and mortality and incidence rate ratios for length of stay) when there is a(n): (1) 10% increase in the proportion of nurses with a BSN degree or higher (nurse education), (2) additional patient-per-nurse, or (3) change in the work environment from poor to mixed or mixed to good. Based on the final models, we plotted predicted probabilities of readmission at varying levels of the nursing resources. Statistical analyses were completed in STATA/MP Version 16.1 (StataCorp LLC, College Station, TX).

## RESULTS

### Patients and Hospital Characteristics

Table 1 presents descriptive characteristics and outcomes of the patient sample. The final analytic sampled included 919,601 surgical patients, of whom 11,610 (1.3%) had OUD. Compared with those without OUD, surgical patients with OUD were younger (mean age of 53.6 vs 63.6; *P* < 0.001), more likely male (50.7% vs 45.5%; *P* < 0.001) and of White race (83.7% vs 79.9%; *P* < 0.001), less likely to be Hispanic ethnicity (11.2% vs 13.4%; *P* < 0.001), disproportionally insured by Medicaid (28.3% vs 10.5%; *P* < 0.001), and to have a patient-directed discharge (3.9% vs 0.5; *P* < 0.001). While surgical patients with OUD more frequently experienced mental health comorbidities such as psychoses (9.2% vs 2.4%; *P* < 0.001) and depression (6.3% vs 4.3%; *P* < 0.001), they less often had physical comorbidities such as hypertension (49.2% vs 58.8%; *P* < 0.001) and renal failure (8.7% vs 10.7%; *P* < 0.001) (Supplementary Table 1, http://links.lww.com/AOSO/A136). Surgical patients with OUD had longer average lengths of stay (8.1 vs 5.1 days; *P* < 0.001), lower rates of 30-day inpatient mortality (0.7% vs 0.9%; *P* = 0.008), and higher rates of 30-day readmission (15.8% vs 9.8%; *P* < 0.001) (Table 1). The most common reasons for readmission for surgical patients with OUD were infection following a procedure (ICD-10 code T814), sepsis (ICD-10 code A419), and acute kidney failure (ICD-10 code N179), respectively.

**Table 1.** Patient Characteristics and Outcomes in Overall Sample and by Opioid Use Disorder (OUD) Status

n (%)	All Patients (n = 919,601)	Patients With OUD (n = 11,610)	Patients Without OUD (n = 907,991)	*P * *†‡
Demographics				
Age (years), mean (SD)	63.3 (16.3)	53.6 (15.2)	63.4 (16.3)	<0.001
Male	418,592 (45.5)	5880 (50.7)	412,712 (45.5)	<0.001
Race				
White	772,583 (79.7)	9606 (83.7)	712,977 (79.7)	<0.001
Black	88,744 (9.8)	1108 (9.7)	87,636 (9.8)	0.695
Asian American/Pacific Islander	28,517 (3.2)	90 (0.8)	28,427 (3.2)	<0.001
Native American	1959 (0.2)	44 (0.4)	1915 (0.2)	<0.001
Other	64,494 (7.1)	626 (5.5)	63,868 (7.1)	<0.001
Hispanic ethnicity	121,123 (13.4)	1279 (11.2)	119,844 (13.4)	<0.001
Transferred in				
Yes	26,160 (2.8)	404 (3.5)	25,756 (2.8)	<0.001
Insurance status				
Medicare	488,165 (53.8)	4914 (43.1)	483,251 (53.9)	<0.001
Medicaid	97,416 (10.7)	3234 (28.3)	94,182 (10.5)	<0.001
Private	271,181 (29.9)	2215 (19.4)	268,966 (30)	<0.001
Other	51,528 (5.6)	1047 (9.2)	50,481 (5.6)	<0.001
Discharge disposition				
Routine	439,965 (47.8)	5004 (43.1)	434,861 (47.9)	<0.001
Post-acute care	461,009 (49.2)	5971 (56.1)	455,038 (50.7)	0.005
Self-directed (against medical advice)	4649 (0.5)	454 (3.9)	4195 (0.5)	<0.001
Died in the hospital	8616 (0.9)	86 (0.7)	8530 (0.9)	<0.001
Other	212 (0.02)	11 (0.1)	201 (0.02)	0.085
Surgical group				
General surgery	326,924 (35.6)	4404 (37.9)	322,520 (35.5)	<0.001
Orthopedic surgery	475,612 (51.7)	6345 (54.7)	469,267 (51.7)	<0.001
Vascular surgery	117,065 (12.7)	861 (7.4)	116,204 (12.8)	<0.001
Elixhauser comorbidities				
Total number, mean (SD)	2.4 (1.9)	3.9 (2)	2.4 (1.9)	<0.001
Outcomes				
Length of stay	5.1 (5.8)	8.1 (11.3)	5.1 (5.7)	<0.001
In-hospital 30-day mortality	8107 (0.9)	76 (0.7)	8031 (0.9)	0.008
30-day readmission	85,291 (9.9)	1727 (15.8)	83,564 (9.8)	<0.001

For discharge disposition, post-acute care was defined as a discharge to home health care, skilled nursing facility, another type of facility, or intermediate care.

*P* values were generated from χ^2^ test for categorical and ANOVA for continuous variables.

*P < 0.05.

†*P* < 0.01.

‡*P* < 0.001.

Descriptive statistics of the overall hospital sample as well as the distribution of surgical patients with and without OUD by hospital characteristics are presented in Table 2. Of note, surgical patients with OUD are seen across hospitals regardless of characteristics such as technology status or nurse educational composition. While there are some statistically significant differences between hospitals where surgical patients with compared with without OUD are cared for, these differences are practically inconsequential (eg, mean of 4.2 vs 4.3 patients-per-nurse, *P* < 0.001; 32.6% vs 31.1% in good compared with mixed or poor work environments, *P* < 0.001).

**Table 2.** Hospital Characteristics in Overall Hospital Sample and Across Patients by OUD Status

n (%)	All Hospitals (n = 448)	Patients With OUD (n = 11,610)	Patients Without OUD (n = 907,991)	*P* *
Bed size				<0.001
Small (≤100)	19 (4.2)	303 (2.6)	14,740 (1.6)	
Medium (101–250)	168 (37.5)	2633 (22.7)	206,300 (22.7)	
Large (>250)	261 (58.3)	8674 (74.7)	686,951 (75.7)	
Teaching status				<0.001
Nonteaching	179 (41.1)	4805 (42)	337,633 (37.8)	
Minor teaching	207 (47.5)	4290 (37.5)	397,787 (44.5)	
Major teaching	50 (11.5)	2356 (20.6)	158,077 (17.7)	
Technology status				0.019
High	266 (60.5)	8403 (73.1)	664,107 (74.1)	
State				<0.001
California	178 (39.7)	4994 (43)	320,665 (35.3)	
Florida	136 (30.4)	4040 (34.8)	299,187 (33)	
New Jersey	86 (19.2)	893 (7.7)	100,849 (11.1)	
Pennsylvania	48 (10.7)	1683 (14.5)	187,290 (20.6)	
Urban				<0.001
Yes	434 (96.9)	11,410 (98.3)	897,104 (98.8)	
% nursing with a BSN or higher				0.4597
Mean (SD)	57.5 (14.5)	60.2 (13.8)	60.1 (14.1)	
Patients per nurse				<0.001
Mean (SD)	4.3 (0.9)	4.2 (0.8)	4.3 (0.8)	
Median (range)	4.2 (2.2–7.9)	4.1 (2.2–7.9)	4.2 (2.2–7.9)	
Nurse work environment				<0.001
Poor	112 (25)	1945 (16.8)	173,963 (19.2)	
Mixed	224 (50)	5878 (50.6)	451,390 (49.7)	
Good	112 (25)	3787 (32.6)	282,638 (31.1)	

Work environment was measured by the PES-NWI excluding the staffing and resource adequacy subscale. Poor are were hospitals in the bottom 25%, mixed work environments are the middle 50%, and good work environments are the top 25% of hospitals.

*P* values were generated from χ^2^ test for categorical and ANOVA for continuous variables.

*P < 0.05.

†*P* < 0.01.

‡*P* < 0.001.

n indicates number.

### Sequential Multivariate Models

Table 3 presents multivariate models showing the association of each nursing resource and postsurgical outcomes. The direct effect model determined the effect of OUD on each outcome. Having OUD was associated with higher odds of readmission, lower odds of mortality, and longer lengths of stay.

**Table 3.** Effects of OUD and Nursing Resources on Readmission, Mortality, and Length of Stay

Variable(s)	Direct Effect Model: OUD Only	Interaction Model 1: OUD × Nurse Education	Interaction Model 2: OUD × Nurse Staffing	Interaction Model 3: OUD × Work Environment
	30-day readmission			
	OR (95% CI)			
OUD	1.73‡ (1.62–1.85)	1.32‡ (0.92–0.96)	1.33‡ (1.24–1.42)	1.24† (1.09–1.42)
Nursing resource	—	0.94‡ (0.92–0.96)	1.03* (1.00–1.05)	0.96† (0.94–0.99)
OUD × nursing resource	—	0.88† (0.81–0.95)	1.09* (1.01–1.18)	1.04 (0.95–1.15)
	30-day in-hospital mortality			
	OR (95% CI)			
OUD	0.74* (0.59–0.93)	0.82 (0.64–1.05)	0.81 (0.63–1.05)	0.96 (0.60–1.54)
Nursing resource	—	0.95 (0.89–1.00)	1.04 (0.99–1.09)	0.88‡ (0.83–0.92)
OUD × nursing resource	—	1.02 (0.71–1.45)	0.97 (0.69–1.35)	0.87 (0.62–1.22)
	Length of stay			
	IRR (95% CI)			
OUD	1.69‡ (1.63–1.76)	1.23† (0.96–0.99)	1.23‡ (1.20–1.26)	1.24‡ (1.18–1.30)
Nursing resource	—	0.98† (0.96–0.99)	1.01 (1.00–1.02)	0.98† (0.96–0.99)
OUD × nursing resource	—	1.01 (0.97–1.05)	1.01 (0.98–1.05)	0.99 (0.95–1.03)

Nursing resource indicates either nurse education, nurse staffing, or work environment depending on the column heading. ORs/IRRs indicate change in risk of outcomes associated with a 10% increase in the proportion of nurses with a BSN degree or higher (for Nurse Education models), each additional patient-per-nurse (for Nurse Staffing models), and each increase in work environment category from poor to mixed and mixed to good (for Work Environment models). Each model accounts for clustering of patients within the 448 hospitals. The Interaction Models control for patient and hospital characteristics. Patient characteristics include age, sex, each Elixhauser comorbidity (except drug abuse), each MS-DRGs, race, ethnicity, insurance status, and discharge disposition. Hospital characteristics include bed size, teaching status, technology status, and urban status. Specific contributions not shown due to space.

— means analysis was not conducted.

*P* values were generated from χ^2^ test for categorical and ANOVA for continuous variables.

*P < 0.05.

†*P* < 0.01.

‡*P* < 0.001.

CI indicates confidence interval; IRR, incidence rate ratio; MS-DRGs, Medicare Severity Diagnosis-Related Groups; n, number.

#### Nurse Education

**FIGURE 2.**
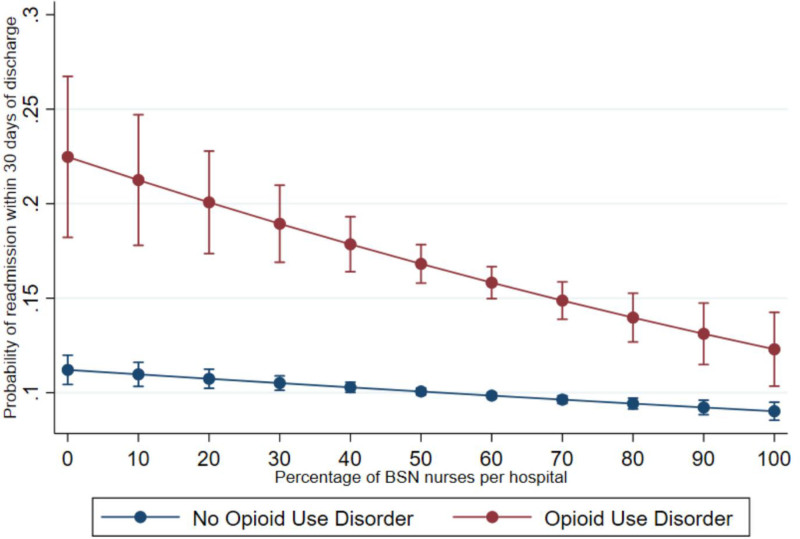
Predicted probability of 30-day readmission for surgical patients with and without opioid use disorder at varying percentages of Bachelor of Science in Nursing nurses. This figure displays the predicted probabilities of 30-day readmission for surgical patients with and without opioid use disorder at varying levels of nurse education.

In interaction model 1 controlling for patient and hospital characteristics (Table 3), we found that each 10% increase in the proportion of BSN-educated nurses was associated with 12% lower odds of 30-day readmission (OR = 0.88; *P* = 0.001) for surgical patients with OUD. To aid in interpretation of the interaction OR, Figure 2 displays the predicted probabilities of 30-day readmission for surgical patients with and without OUD at varying levels of nurse education. This figure suggests that an increase in the hospital-level proportion of BSN nurses from 10% to 80% (80% is the Institute of Medicine recommended level^39^) would be associated with 30 fewer 30-day readmissions per 1000 individuals without OUD but 80 fewer readmissions per 1000 individuals with OUD.

#### Nurse Staffing

**FIGURE 3.**
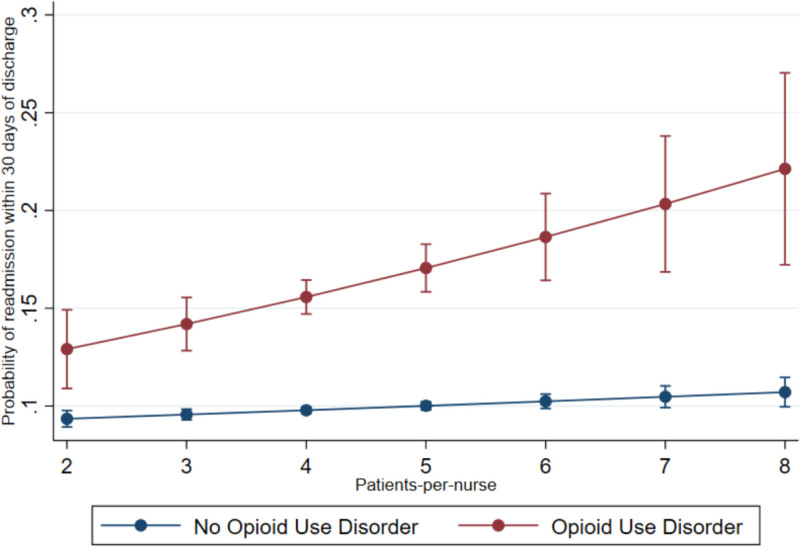
Predicted probability of 30-day readmission for surgical patients with and without opioid use disorder at varying patient-to-nurse staffing levels. This figure displays the predicted probabilities of 30-day readmission for surgical patients with and without opioid use disorder at varying levels of nurse staffing.

Interaction model 2 (Table 3) revealed that each additional patient-per-nurse was associated with significantly higher odds of 30-day readmission (OR = 1.09; *P*=0.024) for surgical patients with OUD. Figure 3 displays the predicted probabilities of 30-day readmission for surgical patients with and without OUD at varying levels of nurse staffing. This figure suggests that an increase in the number of patients-per-nurse from 2 to 8 would be associated with 20 more 30-day readmissions per 1000 individuals without OUD but 90 more readmissions per 1000 individuals with OUD.

#### Nurse Work Environment

Interaction model 3 (Table 3) revealed that each categorical increase in the quality of the work environment (ie, poor to mixed or mixed to good) was associated with significantly lower odds of 30-day readmission (OR = 0.96; *P* = 0.002) and 30-day in-hospital mortality (OR = 0.88; *P* < 0.001), as well as shorter lengths of stay (incidence rate ratio = 0.98; *P* = 0.005) for all surgical patients (including those with OUD) in the sample. The lack of a significant interaction term suggests that the impact of the work environment on surgical patients with OUD is not statistically different from its effect on the overall sample.

## DISCUSSION

Based on a large sample of postsurgical patients undergoing general, orthopedic, or vascular surgeries, we found that the nearly 14,000 patients with OUD experienced longer hospitalizations, lower rates of in-hospital 30-day mortality, and higher 30-day readmission rates than those without OUD. All patients in the sample, including those with OUD, appear to benefit from receiving care in hospitals with better nursing resources, but the effects of nurse education and staffing on surgical patients with OUD were particularly strong. Specifically, each 10% increase in the proportion of nurses educated at the baccalaureate level was associated with twice the odds of 30-day readmission for surgical patients with OUD (12%) compared with 6% for the overall sample. Nurse education was also found to be significantly associated with shorter length of stays for the overall sample. With each additional patient-per-nurse (ie, poorer nurse staffing), odds of 30-day readmission were 3 times higher for surgical patients with OUD (9%) compared with only 3% higher for the overall sample. Consistent with existing evidence,^40,41^ each categorical improvement in the quality of the nurse work environment was associated with improvements in all measured outcomes, although the analysis did not detect a stronger effect of the nurse work environment on postsurgical outcomes for patients with OUD versus those without. Our findings that nursing resources positively impact outcomes for surgical patients with OUD is consistent with a large body of existing evidence on samples of patients including those with Alzheimer’s disease and serious mental illness.^12–14,16,17,20,22^

Surgical patients with OUD have distinct characteristics and require clinical care that is responsive to their unique needs, including pain and withdrawal management.^9,10^ Consistent with other literature, we found that surgical patients with OUD were disproportionally insured by Medicaid^4,42^ and younger,^4^ more frequently experienced mental health comorbidities,^43,44^ and more often had a patient-directed discharge.^45^ Our finding that 1.3% of surgical patients had OUD is higher than another population-based study that found 0.6% prevalence,^5^ but that study used International Classification of Diseases, Ninth Revision codes that are known to be less sensitive for identifying OUD than the ICD-10 codes that we used.^27^ That study^5^ also used data from 2013 to 2014 so we would expect the prevalence to be higher in our later data. The most common reason for readmission for patients with OUD—infection—is consistent with existing evidence.^46^ The findings that patients with OUD more frequently experienced mental health comorbidities such as psychoses (9.2% vs 2.4%; *P* < 0.001) and depression (25% vs 11.5%; *P* < 0.001 are particularly salient given that nurse education and staffing are associated with postsurgical outcomes among patients with comorbid serious mental illness.^16^

Accordingly, surgical patients with OUD require well-organized hospital care that leverages appropriate resources (eg, social work, addiction medicine, pain management). Bedside nurses are key to care coordination^2^ but may only be able to provide and activate appropriate care with the support of adequate nursing resources. Nurses are also key to discharge planning, which is particularly important for these patients who are at elevated risk of a patient-directed discharge and resultant readmission.^11^ It is essential that nurses have adequate time and training to educate patients and provide necessary supports to avoid premature discharge and readmission.

Nursing resources are not static,^22^ and there are examples of purposeful programs to improve them. For example, Magnet accreditation is a formalized approach that many hospitals use to improve nurse work environments.^47^ Literature suggests that Magnet hospitals are differentiated by having better work environments^47,48^ and that the process of undergoing Magnet accreditation over time is associated with improvements in the nurse work environment.^49,50^ Another example of a program to improve the context of nursing care delivery is Transforming Care at the Bedside (TCAB) helps hospitals engage nurses to generate and test ideas to spur practice and process changes that are consistent, efficient, safe, and patient-centered with front-line nursing.^51^ Hospitals involved in TCAB have improved patient safety, patient satisfaction, and cost-savings.^51^

### Implications

Evidence-based public health approaches, including access to naloxone and medications for OUD, have been employed to meet a the needs of people with OUD,^10,52,53^ yet hospitalized surgical patients with OUD continue to suffer adverse outcomes. Our work provides an actionable solution for hospital administrators and policymakers seeking to improve outcomes for surgical patients with OUD: bolster nursing resources. The contribution of this study is the finding that nursing-level factors can be employed to improve the postsurgical outcomes of patients with OUD.

The findings of this study, specifically related to longer length of stay and higher rates of readmission for surgical patients with OUD, have clear cost implications for hospitals. Hospitals may be financially penalized if patients are readmitted, highlighting the importance of leveraging nursing resources to reduce odds of postsurgical readmission. The longer lengths of stay for patients with OUD may result in higher costs for hospitals that are compensated by episodes of care instead of the length of hospitalization. Hospitals seeking to optimize reimbursement should provide high-quality care for surgical patients with OUD, which includes ensuring that nursing resources are adequately supported. Recent evidence on patient-to-nurse ratio policies reveals that 1 additional day in the hospital in poorly staffed hospitals costs hospitals millions of dollars,^54–56^ significant in that surgical patients with OUD are in the hospital for, on average, 3 more days than patients without OUD.

### Limitations

We are unable to make causal inferences due to the cross-sectional nature of our data, although a recent analysis of panel data using data from 2006 and 2016, found that cross-sectional associations of nursing resources with patient outcomes were sustained over time.^22^ Additionally, 30-day in-hospital mortality does not capture deaths that occur following discharge,^9,10^ and persons with OUD are at increased risk for death related to opioid overdose following discharge.^57^ ICD-10 codes may underestimate OUD^58^ but we used an identification approach consistent with existing evidence^5^ and expert consensus. Lastly, the use of illicit fentanyl and other powerful analogues has only worsened since 2016, making pain and withdrawal more difficult to treat and suggesting that the findings from this study are even more important today.

## CONCLUSIONS

Our results about nursing resources and postsurgical outcomes is consistent with decades of evidence showing the importance of having better educated nurses, fewer patients-per-nurse, and strong work environments.^12–14,20,39,59^ Unfortunately, however, variation in nursing resources remains. This study offers an evidence-based solution (ie, bolster nursing resources) to improve outcomes for surgical patients with OUD, an at-risk population that experiences high morbidity, mortality, and high healthcare-related costs.^4,5^ Employing evidence-based and person-centered approaches to care for these patients, like ensuring adequate pain and withdrawal management and connection to medications for OUD as appropriate, is essential^60^ as we seek to optimize care for these patients when they undergo surgery.

## ACKNOWLEDGMENTS

The authors wish to acknowledge Morgan Peele and Jesse Chittams for their analytic support.

## Supplementary Material


