# Supplementary material for: Nursing Resources Linked to Postsurgical Outcomes for Patients With Opioid Use Disorder: An Observational Study
Source: Ann Surg Open. 2022 Jul 22;3(3):e185. doi: 10.1097/AS9.0000000000000185 (PMC9508985; doi:10.1097/AS9.0000000000000185)
Supplement: Supplementary file 1 [file as9-3-e185-s001.pdf]

Supplementary Table 1. Elixhauser Comorbidities in Overall Sample and by Opioid Use Disorder (OUD) Status

n (%)	All patients (n= 919,601)	Patients with OUD (n=11,610)	Patients without OUD (n=907,991)	*p*-value*
**Elixhauser Comorbidities**				
Congestive heart failure	48,452 (5.3)	672 (5.8)	47,780 (5.3)	0.012
Valvular disease	37,773 (4.1)	332 (2.9)	37,441 (4.1)	<0.001
Pulmonary circulation disorders	4,609 (0.5)	91 (0.8)	4,518 (0.5)	<0.001
Peripheral vascular disorders	64,079 (7)	770 (6.6)	63,309 (7)	0.153
Hypertension	539,790 (58.7)	5,710 (49.2)	534,080 (58.8)	<0.001
Paralysis	18,585 (2)	369 (3.2)	18,216 (2)	<0.001
Other neurological disorders	57,570 (6.3)	1,069 (9.2)	56,501 (6.2)	<0.001
Chronic pulmonary disease	151,089 (16.4)	2,826 (24.3)	148,263 (16.3)	<0.001
Diabetes, uncomplicated	135,269 (14.7)	1,041 (9)	134,228 (14.8)	<0.001
Diabetes, complicated	88,531 (9.6)	1,315 (11.3)	87,216 (9.6)	<0.001
Hypothyroidism	125,079 (13.6)	1,389 (12)	123,690 (13.6)	<0.001
Renal failure	98,484 (10.7)	1,011 (8.7)	97,473 (10.7)	<0.001
Liver disease	37,201 (4.1)	1,305 (11.2)	35,896 (4)	<0.001
Peptic ulcer disease	8,774 (1)	158 (1.4)	8,616 (1)	<0.001
AIDS	1,830 (0.2)	91 (0.8)	1,739 (0.2)	<0.001
Lymphoma	4,584 (0.5)	62 (0.5)	4,522 (0.5)	0.584
Metastatic cancer	25,811 (2.8)	236 (2)	25,575 (2.8)	<0.001
Solid tumor without metastasis	14,623 (1.6)	144 (1.3)	14,479 (1.6)	0.002
Rheumatoid arthritis/collagen vascular disorders	30,837 (3.4)	649 (5.6)	30,188 (3.3)	<0.001
Obesity	160,841 (17.5)	2,018 (17.4)	158,823 (17.5)	0.756
Weight loss	36,460 (4)	965 (8.3)	35,495 (3.9)	<0.001
Chronic blood-loss anemia	11,050 (1.2)	167 (1.4)	10,883 (1.2)	0.018
Deficiency anemias	140,049 (15.2)	2,449 (21.1)	137,600 (15.2)	<0.001
Alcohol abuse	32,000 (3.5)	1,221 (10.5)	30,779 (3.4)	<0.001
Drug abuse	22,660 (2.5)	11,610 (100)	11,050 (1.2)	<0.001
Psychoses	22,999 (2.5)	1,066 (9.2)	21,933 (2.4)	<0.001
Depression	105,335 (11.5)	2,906 (25)	102,429 (11.3)	<0.001
Coagulopathy	39,397 (4.3)	736 (6.3)	38,661 (4.3)	<0.001
Fluid and electrolyte disorders	163,554 (17.8)	2,984 (25.7)	160,570 (17.7)	<0.001

Note: *P*-values were generated from χ^2 for categorical and ANOVA for continuous variables. *p-value <0.05; **p-value <0.01; ***p-value <0.001.
